# Hepatic falciform ligament clear cell myomelanocytic tumor: A case report and a comprehensive review of the literature on perivascular epithelioid cell tumors

**DOI:** 10.1186/s12885-015-1992-4

**Published:** 2015-12-23

**Authors:** Zu-Sen Wang, Lin Xu, Lin Ma, Meng-Qi Song, Li-Qun Wu, Xuan Zhou

**Affiliations:** Department of Hepatobiliary Surgery, Affiliated Hospital of Qingdao University, Qingdao, Shandong 266003 China; Department of General Surgery, Qingdao Eighth People’s Hospital, Qingdao, Shandong 266003 China; Department of Pathology, Affiliated Hospital of Qingdao University, Qingdao, Shandong 266003 China

**Keywords:** CCMMT, Diagnosis, Immunohistochemistry, PEComa

## Abstract

**Background:**

The objective of the study was to explore the clinical expression, radiological and pathological features, differential diagnosis, and biological behavior of a clear cell myomelanocytic tumor. In a case involving a clear cell myomelanocytic tumor located in the hepatic falciform ligament, we evaluated clinical expression, radiological characteristics, histopathology, immunohistochemistry, and biological behavior; we also reviewed the relevant literature.

**Case presentation:**

Clear cell myomelanocytic tumor is a benign soft-tissue neoplasm that often occurs in women, and is expressed as a painless mass. The falciform ligament is its most frequent site of occurrence. The imaging characteristics of this lesion were uneven enhancement in the arterial phase, continuing to strengthen in the venous phase, and equal density in the balance phase. Histological and immunohistochemical analysis revealed the main transparent epithelioid cells and smooth muscle spindle cells to be HMB-45(+), smooth muscle actin(+), and melan-A (+).

**Conclusion:**

Hepatic vascular epithelioid cell tumors are very rare mesenchymal neoplasms. Few studies have investigated this tumor in the hepatic falciform ligament; consequently, its diagnosis and the selection of an appropriate treatment and follow-up protocol are challenging. Treatment outcome remains unpredictable. Therefore, clear cell myomelanocytic tumor should be viewed as a tumor with uncertain malignant potential requiring long-term follow-up.

## Background

Perivascular epithelioid cell (PEComa) tumor has recently been cytopathologically defined. Histological and immunohistochemical analysis indicate that it has the obvious characteristics of perivascular epithelioid cells (PECs) [1, 2]. A type of tumor with hyaline cells that has the characteristics and similarity to a neoplasm with perivascular epithelioid cell differentiation is determined as follows according to the World Health Organization (2002) soft-tissue tumor classification: a neoplasm with perivascular epithelioid cell differentiation, including hepatic falciform ligament clear cell myomelanocytic tumors [3]. Because soft-tissue clear cell myomelanocytic tumor (CCMMT) is a newly identified tumor type, there have been very few previous studies. This tumor usually involves the uterus, followed by the sickle ligament and gut. There have been only two reported cases of CCMMT in the liver [4]; thus, the diagnosis and differential biological behavior of this neoplasm require further study. The present report involves the evaluation of a case of CCMMT and a review of the relevant literature.

### Case presentation

A 29-year-old woman was admitted to our hospital in July 2014 with liver cancer rupture after intervention over a period of 1 month. She had a treatment history involving L-carnitine drugs proceeded by cesarean section (4 months ago), and no history of hepatitis and hepatocirrhosis. A physical examination revealed the following: the right side of the abdomen was slightly bloated; there was a 10-cm surgical scar on the hypogastrium, and a palpable 5 × 6 cm mass was present in the right upper abdomen; the mass was hard, smooth, and had good texture; and there was no pain when the mass was pressed. Laboratory examination revealed routine hepatic and renal function, and normal levels of serum electrolytes and alpha fetoprotein.

**Fig. 1:**
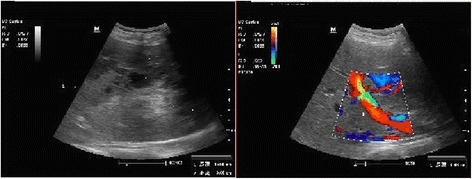
Ultrasound scan of the digestive system. A heterogeneous high echogenic mass can be seen in the right liver lobe. An irregular anechoic area is also evident in the mass; color Doppler flow imaging showed no obvious blood signal in it

**Fig. 2:**
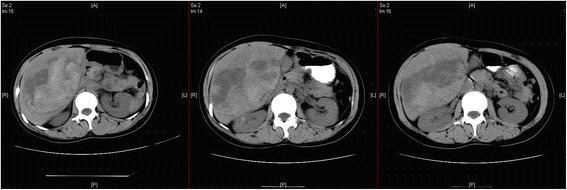
Upper abdominal computed tomography scan. A giant tumor can be seen in the right anterior superior segment of the liver. Its internal density is uneven and exhibits a high/low/equal mix

Ultrasound examination of the digestive system demonstrated the following: a heterogeneous hyperechoic mass (15.5 × 9.6 × 14.2 cm) located in the right hepatic lobe; an irregular anechoic area in the mass; a clear boundary; and no obvious blood flow signal in the mass examined using color Doppler flow imaging (Fig. 1). A plain abdominal computed tomography (CT) scan showed a huge abdominal mass below the right segment of the anterior hepatic lobe, an uneven internal density, and high and low mixed density (Fig. 2). A dynamic enhanced CT scan of the upper abdomen demonstrated the following: a mixed high- and low-density mass shadow in the right anterior hepatic lobe below segment S5; a clear boundary; a neat outline; significant uneven enhancement; internally, a large area of low-density non-enhanced shadow; multiple enlargement of the surrounding vessels; and a low-density coated edge.

**Fig. 3:**
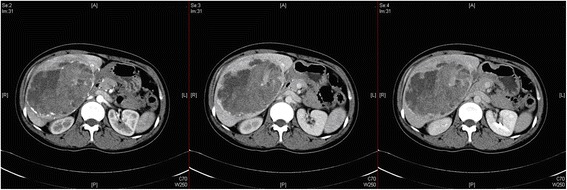
Upper abdominal dynamic enhancement computed tomography scan. Hepatic arterial enhancement is uneven, but the strengthening of the venous and balance periods is more even

**Fig. 4:**
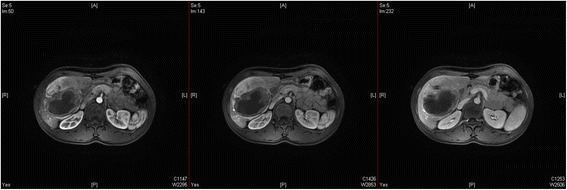
Magnetic resonance imaging scan of the upper abdomen. The mass exhibits uneven strengthening and the internal liquefied necrotic area has no strength

Hepatic arterial enhancement was uneven, but the venous and balance periods strengthened more evenly (Fig. 3). A dynamic enhanced magnetic resonance imaging (MRI) scan of the upper abdomen revealed the following: a huge mass in the right anterior hepatic lobe; an uneven internal density; an obvious uneven strengthening; and a visible liquefied necrotic area within the mass (Fig. 4). A hepatic adenoma was diagnosed using the imaging data. During surgery, the following was found: the mass was located in the V segment of the liver; its diameter was approximately 10 cm; and the mass exhibited cystic and solid characteristics, and had invaded the gallbladder and part of the hepatic segment VI. Consequently, the patient underwent hepatic V segment excision, partial VI segment excision, and gallbladder excision. Postoperative recovery was good.

#### Pathological examination

##### Macroscopic examination

Macroscopic examination revealed the following: a liver tissue of size 19 × 14 × 7 cm; the presence of a nodular goiter in the liver after sectioning with an area of 11 × 9 cm; the goiter was grayish red and grayish yellow and had a crisp quality; and there was partial invasion of the local liver capsule.

##### Microscopic examination

**Fig. 5:**
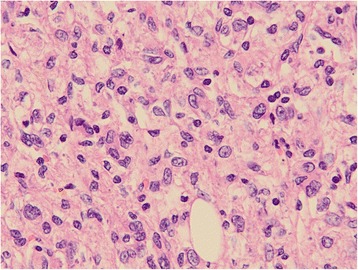
Microscopic examination. Tissue sections after hematoxylin-eosin staining were observed at 400× magnification

Microscopic examination revealed the following: the tumor had a high cell density; cells were fusiform or ovoid in shape; in parts of the tumor, the cells were epithelioid; the cytoplasm was weakly acidophilic or bright; small nucleoli and nuclear grooves were visible; mitotic figures were rare; there was a diffuse distribution of tumor cells; the lesion had an obscure boundary; a hemorrhage was present in part of the tumor; necrosis was evident; and a multinucleated giant cell reaction occurred. Liver cell edema associated with mild cholestasis was observed in the surrounding hepatic tissues, together with dilated vessels in the central vein and portal area (Fig. 5).

#### Immunohistochemistry

**Fig. 6:**
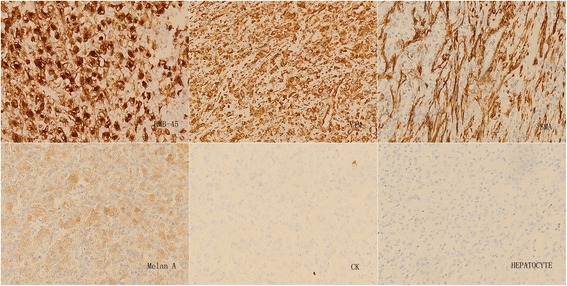
Immunohistochemistry 1. Immunohistochemical analysis of tumor cells indicated the following: HMB-45(+), vimentin(+), smooth muscle actin (SMA)(+), melan-A(+,CK(−), and hepatocyte(−)

**Fig. 7:**
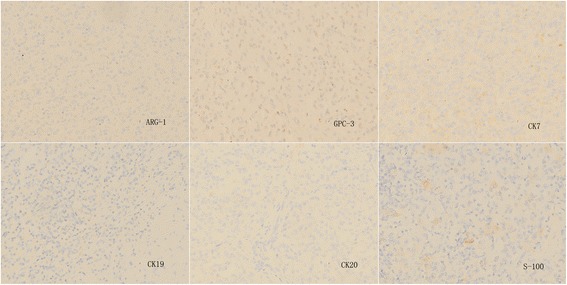
Immunohistochemistry 2. Immunohistochemical analysis of tumor cells indicated the following: Arg-1(−), GPC-3(−), CK7(−), CK19(−), CK20(−), and S-100 (−)

Immunohistochemical analysis of tumor cells indicated the following: HMB-45(+); vimentin(+); smooth muscle actin (SMA)(+); melan-A(+); CK(−); hepatocyte(−); Arg-1(−); GPC-3(−); CK7(−); CK19(−); CK20(−); S-100(−); CD34 blood vessel endothelium(+); and no exact tumor suppository (Figs. 6 and 7). The pathological diagnosis was a hepatic falciform ligament clear cell myomelanocytic tumor.

## Discussion

PEComa is a tumor family that includes the following: angiomyolipoma (AML), a clear cell “sugar” tumor of the lung (CCST); lymphangioleio-myomatosis (LAM), CCMMT with rare occurrence in the pancreas; and lucency cell tumors in the rectum, peritoneum, uterus, and other organs. The other tumors occur in many parts of the body, including the chest, gastrointestinal tract, sinus, bone trunk, and liver; most have been described exclusively in case reports [5–8]. In 1992, Bonetti et al. [9] first proposed the name lung hyaline cell “sugar” tumor for angioleiomyolipoma and lymphangioleiomyomatosis cells with the same morphology and immune phenotype as PECs. In 1996, Zamboni et al. [10] reported a case of lung hyaline cell “sugar” tumor, which was very similar to pancreas hyaline cell tumor; consequently, we suggested the term PEComa for PEC tumors located in different places and with no connection. Armah et al. [11] proposed that PEComa tumors, with the exception of AML, LAM, and CCST, should be named PEComa-NOS (not otherwise specified) tumors, or should be known by some other name such as CCMMT or primary extrapulmonary sugar tumor; they used monotype epithelioid AML as a synonym of PEComa. The name PEC sarcoma has been changed to PEComa. CCMMT is one of the tumors originating from blood vessels surrounding epithelioid cells. In our case, the CCMMT in the falciform ligament belonged to the PEComa family.

### Clinical characteristics

Jafari et al. [12] reported the following: hepatic PEComa occurred more often in women; the peak incidence was in patients aged 40–70 years; it occurred more frequently in the liver; many of the tumors were solitaryand easily misdiagnosed as hepatocellular carcinoma; and a few tumors exhibited malignant behavior. The onset of the disease manifests insidiously, and most patients have no history of liver diseases. The symptoms and signs of the disease reveal no specificity; furthermore, they are similar to other tumors arising from the liver. Generally, indigestion, loss of appetite, nausea, and intermittent colic pain can occur. On physical examination, tenderness to palpation and liver enlargement may be observed; in some patients, these tumors have been detected by means of imaging examination during a health check-up. The tumor markers AFP, CEA, and CA19-9 were all negative. Most patients were diagnosed after a postoperative pathological examination [13, 14]. Priola et al. [15] reported a case of acute abdominal disease caused by PEComa.

### Imaging manifestations

Imaging manifestations of hepatic PEComa have rarely been mentioned in previous studies. Hepatic PEComa exhibits a variety of imaging findings. Ameurtesse et al. [16] found that hepatic PEComa could be expressed as a range of echo types under ultrasound examination, and, in most of the conditions, the lesions or tissue surrounding the lesions expressed a rich blood supply. Generally in the CT or MRI scans, the lesions in the arterial phase strengthen more obviously, continue to strengthen in the venous phase; delayed scanning usually reveals equidensity [17–19].

Most of the lesions show low signal intensity in T1-weighted imaging, and high signal intensity in T2-weighted imaging; a rich tumor blood supply is usually associated with liver cancer and liver hemangioma [20]. Preoperative imaging studies (CT and MRI) have poor diagnostic sensitivity regarding PEComa; the reported preoperative diagnostic accuracy of CT and MRI is 15.7 % (11/70) and 22.7 % (10/44), respectively [21]. Imaging findings regarding our case were basically the same as those previously reported. The lesion had a liquefied necrotic area, strengthening of the liver parenchyma in the arterial phase was uneven, and strengthening in the intravenous and balance periods were even. The preoperative diagnosis was hepatic adenoma, and postoperative pathology revealed CCMMT located in the hepatic falciform ligament. There have been numerous reports regarding AML imaging, but reports concerning the imaging features of CCST, LAM, and CCMMTs are rare; preoperative imaging diagnosis is difficult and mainly relies on postoperative pathologic examination.

### Pathological features and immunohistochemistry

#### Pathological characteristics

PEComa tumor cells always surround blood vessels and are arranged in a radiated or sleeve pattern. Usually, the adjacent blood vessel cells are epithelioid in shape; cells that are located far from the blood vessels have a fusiform shape and the appearance of smooth muscle cells. The cytoplasm of the tumor cells is clear and eosinophilic [22], the nucleus is usually small and round or oval in shape, has a small nucleolus, contains fine chromatin, is located in the meso-position, and nuclear division is rarely seen. However, in a few cases it is possible to observe obvious hyperchromatic irregular-shaped nuclei, an increase in the size of the nucleolus, an increase in the number of mitotic figures, the presence of melanophores, focal necrosis, and an intravascular tumor embolus; these findings indicate a poor prognosis [23–25]. PEComa-NOS and PEComa occurring in different regions of the body are similar. The lesion can be formed by epithelioid PECs and is referred to as CCST like; it can also be formed by fusiform PECs, and usually appears similar to the funicular line that is referred to as CCMMT like.

#### Immunohistochemistry

PEComa tumors have similar immunohistochemical characteristics, mainly including melanin cell markers (HMB-45 and/or melan A) and smooth muscle cell markers (microfilament protein and/or desmin) [22]. In 1991, two studies reported that hepatic and kidney PEComa tumors were all positive for HMB-45 [26, 27]. These findings were confirmed in the present study. Melanocytes and smooth muscle cell markers play a significant role in the diagnosis of this type of tumor [28, 29]. In previous studies, it was believed that PEComa tumors simultaneously expressed melanocyte and smooth muscle cell markers. However, a study by Folpe et al. demonstrated that only 80 % of cases simultaneously expressed these two types of markers [6]. Thus, negativity for smooth muscle cell markers did not rule out the diagnosis of having this disease [30]. Ameurtesse et al. [16] concluded that the immunohistochemical characteristics of hepatic PEComas included: generally positive expression of HMB-45; frequently positive expression of melan-A and SMA; and negative expression of S100, desmin, and vimentine. With the exception of the immunohistochemical markers detailed above, the expression of other markers including CD34 and CD117 differed between PEComas [18, 31, 32].

### Differential diagnosis

The differential diagnosis of CCMMT relies predominantly on cell morphology and immunohistochemistry [33].

#### Soft-tissue clear cell sarcoma

Both soft-tissue clear cell sarcoma and CCMMT occur more frequently in dense connective tissue, have common clear cells and nucleoli, visible melanin particles, and the tumor cells express HMB-45; thus, they are easily confused. However, soft-tissue clear cell sarcoma always occurs in limb tendons and the tendon membrane area. It has the following characteristics: the cells are fusiform; it typically has a nest-like distribution; the nucleolus is bigger and more obvious than those in CCMMT cells; it shows strong basophilia; and it has a lack of characteristic vascular components [34]. Immunohistochemistry may be valuable in distinguishing between these two tumor types. CCMMT co-expresses melanocytic markers and smooth muscle markers but lack S-100 protein expression, whereas clear cell sarcoma in the tendons and aponeuroses express both S-100 protein and the melanocytic markers, but do not express smooth muscle actin or myosin [35, 36].

#### Malignant clear cell mesothelioma

All malignant clear cell mesotheliomas occur in the abdominal cavity and have clear cells. However, malignant clear cell mesothelioma exhibits the following characteristics: malignant progression, always with ascites; a tendency to grow in the peritoneum; a nodular-like appearance; the presence of clear cells that are partially gland like; the edematous degeneration of papilloma epithelial cells; lipid accumulation in foam sample cells can occur; and immunohistochemically it is positive for CK and Vim. Its positivity for the mesothelial cell-related antibody allows it to be distinguished from CCMMT cells [33, 37].

#### Clear cell leiomyoma

Clear cell leiomyoma cells are similar to CCMMT clear cells and smooth muscle spindle cells, and have comparable positive expression of SMA; however, CCMMT cells have a lighter nucleus, its entire cytoplasm appears clear, vacuoles in some cells compress the nucleus and show a signet ring shape, and the epithelioid cells all exhibit a funicular distribution. Clear cell leiomyoma cells do not express HMB-45, and can therefore also be distinguished from CCMMT cells [38].

#### Dedifferentiated liposarcoma

The key factor in the diagnosis of dedifferentiated liposarcoma is to determine if the tumor contains true lipoblasts; liposarcoma can also express S100 protein, but not HMB-45 [39].

#### Leiomyosarcoma

Leiomyosarcoma differs from CCMMT in that it displays shorter fascicles that intersect at right angles to one another, more pronounced cytoplasmic eosinophilia,and elongated nuclei with blunt ends [33]. The nested growth pattern, the vesicular nuclei with small prominent nucleoli, and the rather uniform clear cell change that characterize CCMMT would not usually be expected to be encountered in leiomyosarcoma. In addition, leiomyosarcoma usually does not express antibodies to melanosomal proteins, such as S-100 or melan-A [40, 41]. Additionally, the majority of leiomyosarcomas express desmin [42], in contrast to CCMMTs, which are desmin negative.

#### Monotypic angiomyolipoma

Recently, monotypic angiomyolipomas which differ from the usual angiomyolipoma have been described. They consist entirely or nearly entirely of perivascular epithelioid cells with only rare thick-walled blood vessels or areas of “adipocytic differentiation”. These tumors differ significantly from CCMMTs because they are composed of epithelioid cells and exhibit a lack of the spindled cells with cytoplasmic clearing and the characteristic nested growth pattern of CCMMT [43, 44]. CCMMT should also not be confused with malignant angiomyolipoma. These tumors differ from CCMMTs in that they show sarcomatous differentiation with marked nuclear pleomorphism, elevated mitotic activity, and necrosis [1, 46].

Other common differential diagnoses include: epithelioid sarcoma; hepatic adenoma and carcinoma; gastrointestinal stromal tumors; metastatic sarcomatoid renal cell carcinoma; paraganglioma; and oncocytic and clear cell carcinoma. The positivity for melanocytic markers and the negativity for multiple markers including CK, CD34, S-100, and EMA confirm the diagnosis (Table 1) [22, 14, 16].

**Table 1:** Immunohistochemistry in the differential diagnosis of CCMMT

	CCMMT	Hepatic carcinoma	Melanoma	Gastrointestinal stromal tumors	Paraganglioma
SMA	positive			variable	
HMB-45	positive		positive		
Melan-A	positive		positive		
S-100	negative		positive	variable	positive
CK	negative	positive	negative	negative	
EMA	negative	positive	negative		
CD34	positive			positive	

### Biological behavior

Blood vessels surrounding the epithelioid cells in the tumor are characteristic of benign and malignant undefined tumors. Most patients with PEComas demonstrate benign biological behavior and unfavorable prognosis, while a few have malignant behavior and an unfavorable prognosis [6, 46]. At present, there are no clear diagnostic criteria for malignant PEComa; the clinical biological behavior of the tumor has always been controversial. According to the World Health Organization 2003 guidelines, a PEComa tumor should be viewed as malignant if it exhibits the following features: infiltrating growth; a high cell density, nuclear enlargement and hyperchromatism; an increased number of mitotic figures; and atypical nuclear division and coagulative necrosis are present. In 2005, Flope et al. [6] studied 26 cases of PEComa that occurred in the soft tissue and gynecologic reproductive organs; they proposed a series of standards whereby it could be subdivided into tumors having benign characteristics, uncertain malignant potential, or malignant potential.

Diagnostic criteria for malignant PEComa should include two or more of the following: tumor size >5 cm; infiltrative growth; a high nuclear and cell density; a mitotic ratio ≥1/50 high power; and signs of coagulative necrosis and vascular invasion. PEComa of uncertain malignant potential only exhibits polymorphism/multicore giant cells, or only has a size >5 cm, but no other histological abnormalities. Benign PEComa tumors are <5 cm in size and have no other histologic abnormalities. These criteria have gradually been applied in PEComa diagnosis, but still have limitations, and there are special cases were they are not applicable; for example, in long-term lymphangioleiomyomatosis involving a wide range, the influence of pulmonary interstitial fibrosis caused by the disease is similar to the effects of a tumor. The recurrence and metastasis of epithelioid PEComa is apparently higher than is the case in normal PEComa. Thus, an accurate assessment of the biological behavior of PEComa and prognosis data accumulated from more cases and clinical long-term follow-up studies will be required.

### Treatment and prognosis

Currently, excision is the only method that has been used to treat this disease, and can achieve a radical cure in most cases. Although most PEComa tumors exhibit benign behavior, there have been reports of local invasion and remote metastasis [47, 48]. In addition, there have been a few reports of remote metastasis in post-excision cases [48–50]. However, no other effective method is currently available for the treatment of malignant PEComa, especially post-operation. In a study by Martignoni et al. [1], it was shown that activated mTORC1 to TSC, related or not related to PEComa, had an important function; an mTORC1 inhibitor such as rapamycin may have a therapeutic effect regarding PEComa. In an animal TSC model study that preceded the preclinical phase study, a significant curative effect was demonstrated; the same treatment protocol was also effective in kidney AML. Wagner et al. [51] evaluated oral administration of the mTOR inhibitor sirolimus in the treatment of three cases of malignant PEComa; the imaging data revealed that the tumor responded to treatment, indicating that it could potentially be used as a targeted therapy for PEComa. Italiano et al. [52] reported cases where the mTOR inhibitor temsirolimus had the same effect; however, more clinical trials are required to confirm this finding. Long-term close follow-up of patients with PEComa is necessary, and PET-CT can be used as part of the follow-up examination protocol [53, 54].

## Conclusions

An epithelioid cell tumor surrounded by hepatic blood vessels is a very rare gastrointestinal mesenchymal tumor; a hepatic falciform ligament CCMMT belongs to this group. Relevant studies regarding the methods used for its diagnosis are rare.

Treatment methods and questions concerning the design of the follow-up protocol remain a challenge, and prognosis continues to be unpredictable. All of these lesions should be diagnosed as tumors with uncertain malignant potential, which require stricter long-term follow-up evaluation.

### Ethics Statement

The study was approved by the Ethic Committee of the Affiliated Hospital of Qingdao University Medical College. The IRB number is QYFYEC KY2015-002-010. The patient provided written informed consent.

### Consent

Written informed consent was obtained from the patient for publication of this Case report and any accompanying images. A copy of the written consent is available for review by the Editor of this journal.

## Abbreviations

PEComa: Perivascular epithelioid cell tumor
PECs: Perivascular epithelioid cells
CCMMT: Clear cell myomelanocytic tumor
CT: Computed tomography
MRI: Magnetic resonance imaging
HMB-45: Human melanoma black 45
SMA: Smooth muscle actin
melan-A: Protein melan-A
CKs: Cytokeratins
EMA: Epithelial membrane antigen
Arg-1: Arginase 1
GPC-3: Glypican 3
CD34: Cluster of differentiation antigen 34
